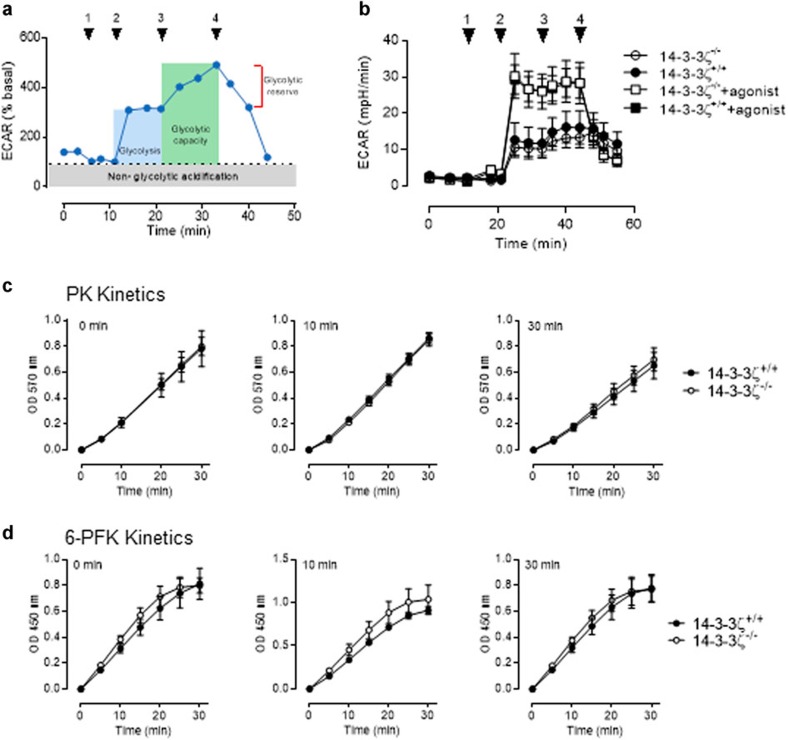# Corrigendum: 14-3-3ζ regulates the mitochondrial respiratory reserve linked to platelet phosphatidylserine exposure and procoagulant function

**DOI:** 10.1038/ncomms16125

**Published:** 2017-08-30

**Authors:** Simone M. Schoenwaelder, Roxane Darbousset, Susan L. Cranmer, Hayley S. Ramshaw, Stephanie L. Orive, Sharelle Sturgeon, Yuping Yuan, Yu Yao, James R. Krycer, Joanna Woodcock, Jessica Maclean, Stuart Pitson, Zhaohua Zheng, Darren C. Henstridge, Dianne van der Wal, Elizabeth E. Gardiner, Michael C. Berndt, Robert K. Andrews, David E. James, Angel F. Lopez, Shaun P. Jackson

Nature Communications 7: Article number: 12862; DOI: 10.1038/ncomms12862 (2016); Published: 09 27 2016; Updated: 08 30 2017

In Supplementary Fig. 7 of this Article, graphs presenting 6-PFK kinetics in panel d were inadvertently duplicated from those in panel c. The correct version of Supplementary Fig. 7 appears below as Fig. 1.

## Figures and Tables

**Figure 1:**