# Supplementary material for: 14-3-3ζ regulates the mitochondrial respiratory reserve linked to platelet phosphatidylserine exposure and procoagulant function
Source: Nat Commun. 2016 Sep 27;7:12862. doi: 10.1038/ncomms12862 (PMC5052641; doi:10.1038/ncomms12862)
Supplement: Supplementary Information — Supplementary figures 1-8 [file ncomms12862-s1.pdf]

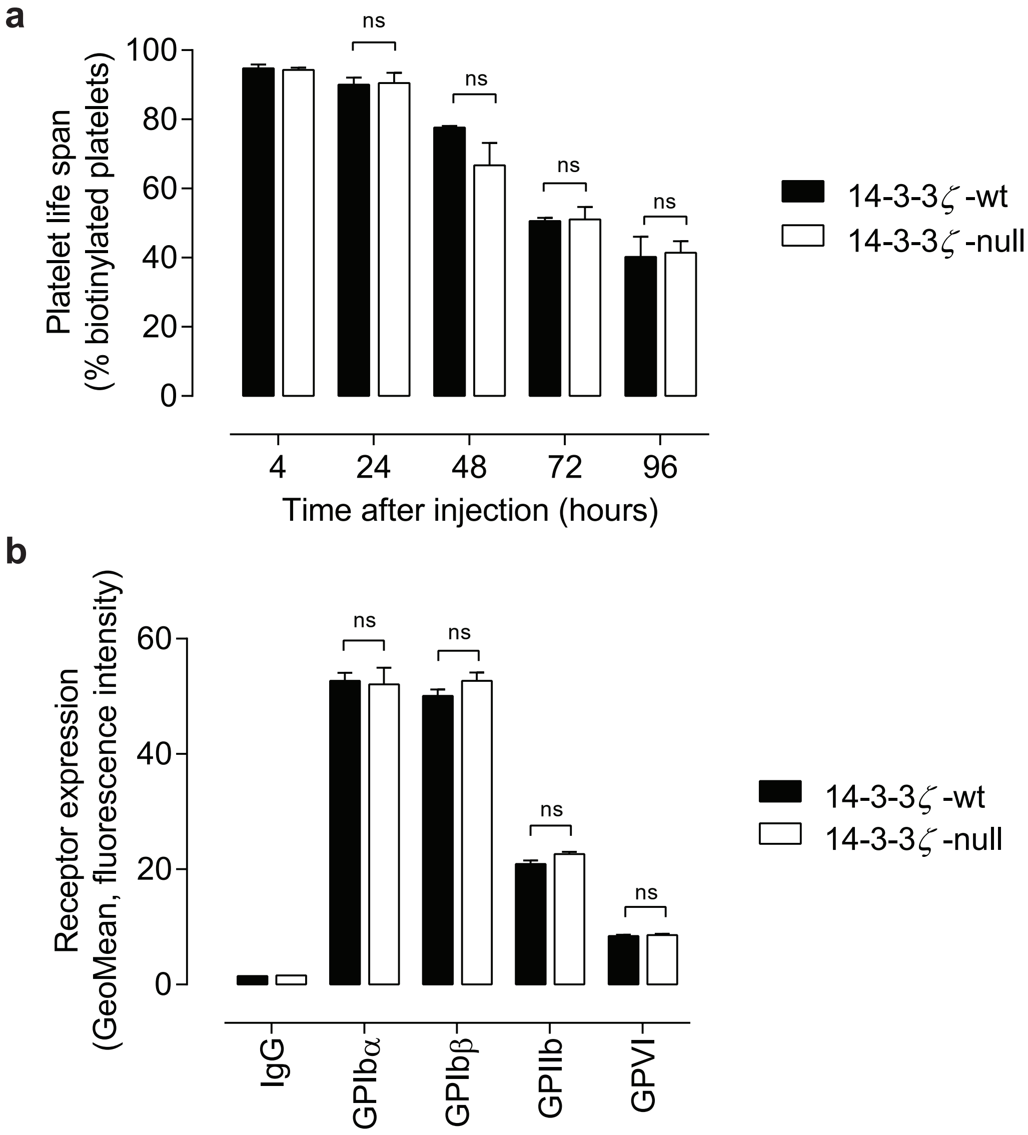

Supplementary figure 1.

Platelet life span and surface adhesion receptors expression in unaltered in 14 3 3ζ-deficient mice.
14-3-3ζ-wt and 14-3-3ζ-deficient (14-3-3ζ-null) were examined for (a) Platelet life span (n = 4) and (b) receptor expression [14-3-3ζ-wt n=11; 14-3-3ζ-null n=12], as described under “Materials and Methods”. Results were analyzed using two-way ANOVA with Bonferroni’s post-hoc test.

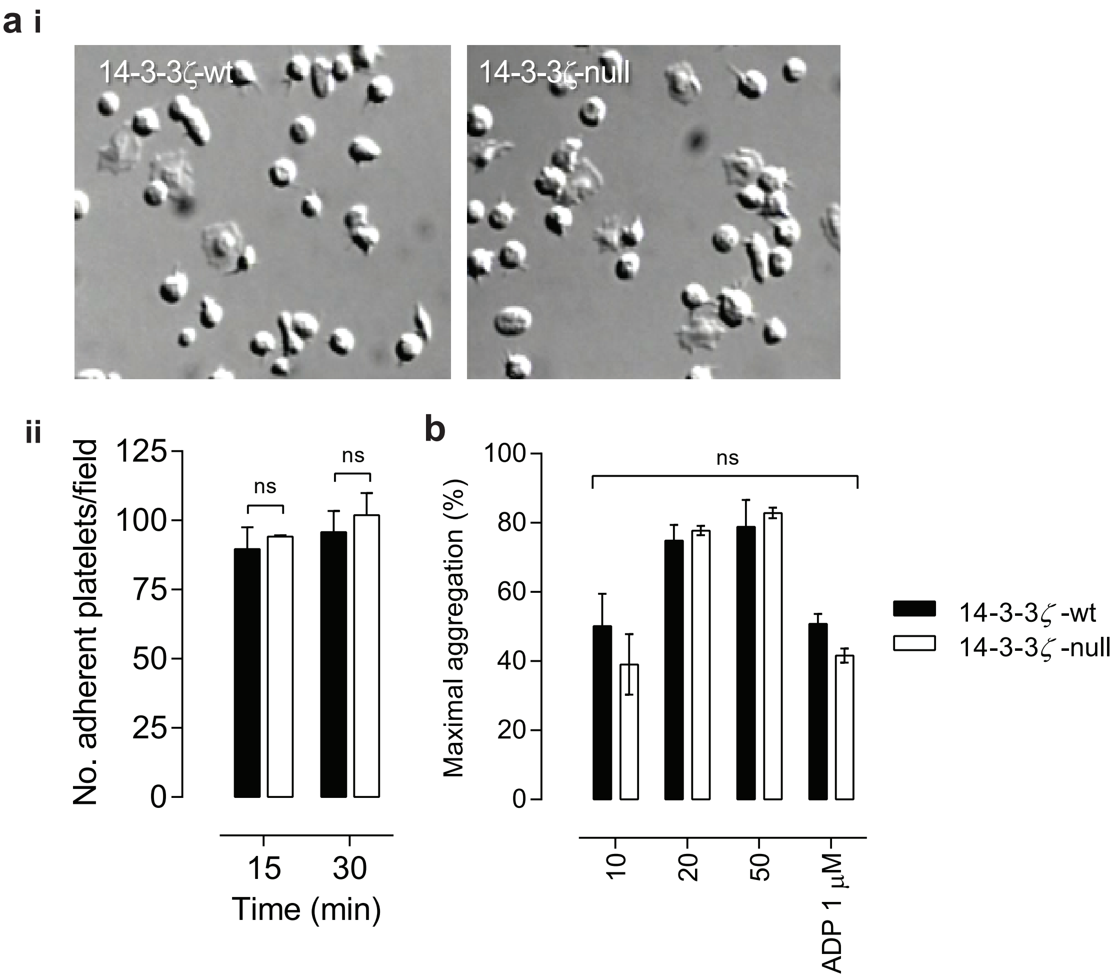

Supplementary figure 2.
GPIbα-VWF adhesion and platelet aggregation are unaffected in 14 3 3ζ-deficient mouse platelets.
(a) Adhesion of 14-3-3ζ-wt or 14-3-3ζ-deficient (14-3-3ζ-null) platelets (2×10^7 ml^{-1}) to human VWF (50 μg ml^{-1}) in the presence of botrocetin (10 μg ml^{-1}) was examined, as described under "Methods". Platelet adhesion was imaged by DIC microscopy (Leica DMIRB, water immersion objective: x 63, NA: 1.2) and images captured using DVT tools (Pinnacle Systems, USA). (i) Images are taken from one experiment, representative of 5 independent experiments, with the histogram (ii) depicting the number of adherent platelets per field (mean ± SEM, n=5). (b) Aggregation of washed platelets in response to CRP or ADP was compared between 14-3-3ζ-wt or 14-3-3ζ-null mice. This histogram depicts the mean +/- SEM of 3 independent experiments. (a,b) Results were analyzed using a 2-way ANOVA (Bonferroni's post-hoc testing).

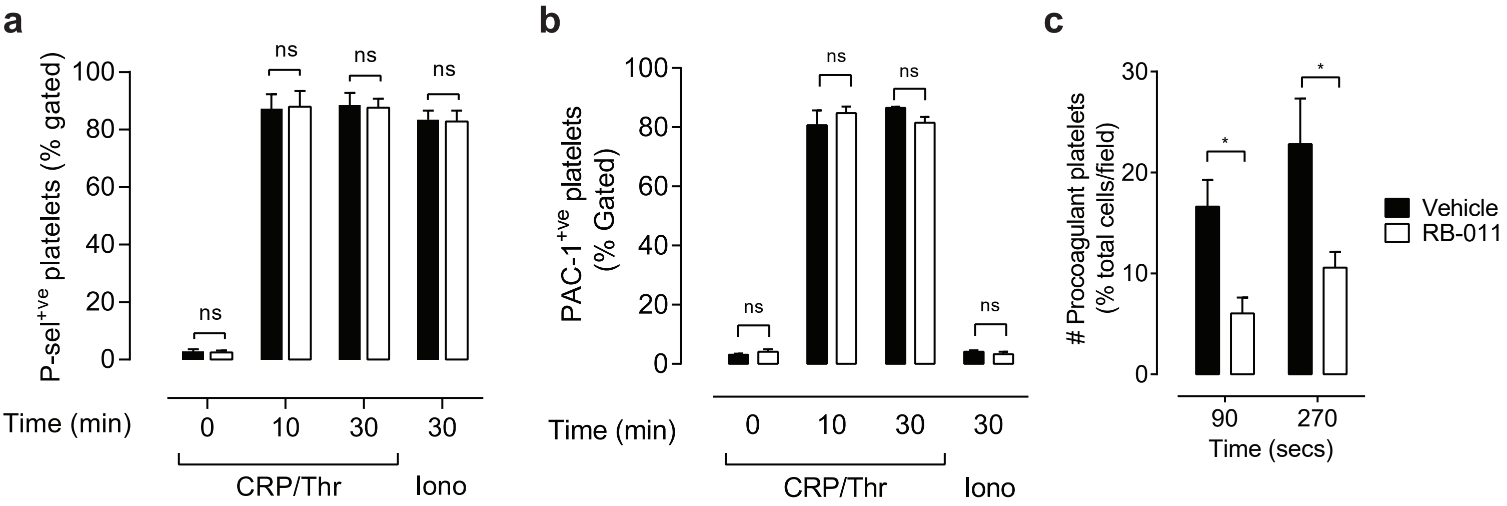

Supplementary figure 3.

The 14-3-3 dimer destabiliser reduces platelet procoagulant function under flow conditions.
Washed platelets were isolated from anticoagulated whole blood from healthy volunteer donors and treated with a 14-3-3 dimer destabiliser (RB-011, 10 µM) or vehicle (sodium mesylate salt). (a) Washed platelets were perfused into Type I collagen (250 μg ml^{-1}) coated microslides at 300 s^{-1}, and allowed to settle for 5 minutes in the absence of flow. Development of procoagulant platelet morphology was assessed under flow conditions (300 s^{-1}) over time using DIC microscopy [Leica DMIRB, water immersion objective: x63, NA 1.2] and images recorded for off-line analysis using Image J. The number of procoagulant platelets was expressed as a % of the total number of platelets per field. Results are expressed as the mean ± SEM (n=3), and analysed using a 2-way ANOVA (Bonferroni's post-hoc testing) where *$p<0.05$. (b) P-selectin and (c) integrin $\alpha_{IIb}\beta_3$ activation in response to the indicated concentrations of agonist were quantified through measurement of FITC-conjugated P-selectin (P-sel^{+ve}) or PAC-1 antibody binding, respectively, as described under "Methods".

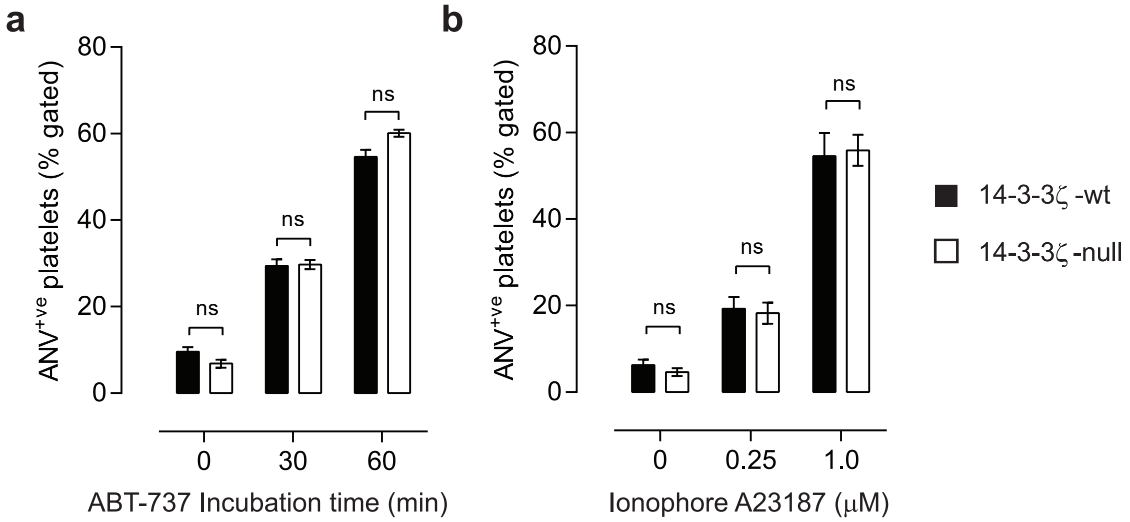

Supplementary figure 4.
PS exposure in response to apoptosis or calcium ionophore are normal in 14-3-3ζ-deficient platelets. Diluted whole blood samples from 14-3-3ζ-wt (black bars) or 14-3-3ζ-deficient (14-3-3ζ-null, blue bars) mice were treated with (a) ABT-737 [1 μM, indicated times, n=3-6] or (b) calcium ionophore A23187 [30 min, n=10], in the presence of Alexa-488-labelled Annexin V (ANV). Each figure represents the percentage (%) of platelets positive for Annexin V (ANV+ve platelets). Results are expressed as mean ± SEM from the indicated number of independent experiments (a: n=3; b: n=5), and analysed using a 2-way ANOVA (Bonferroni's post-hoc testing), where $^{ns}p>0.05$. where

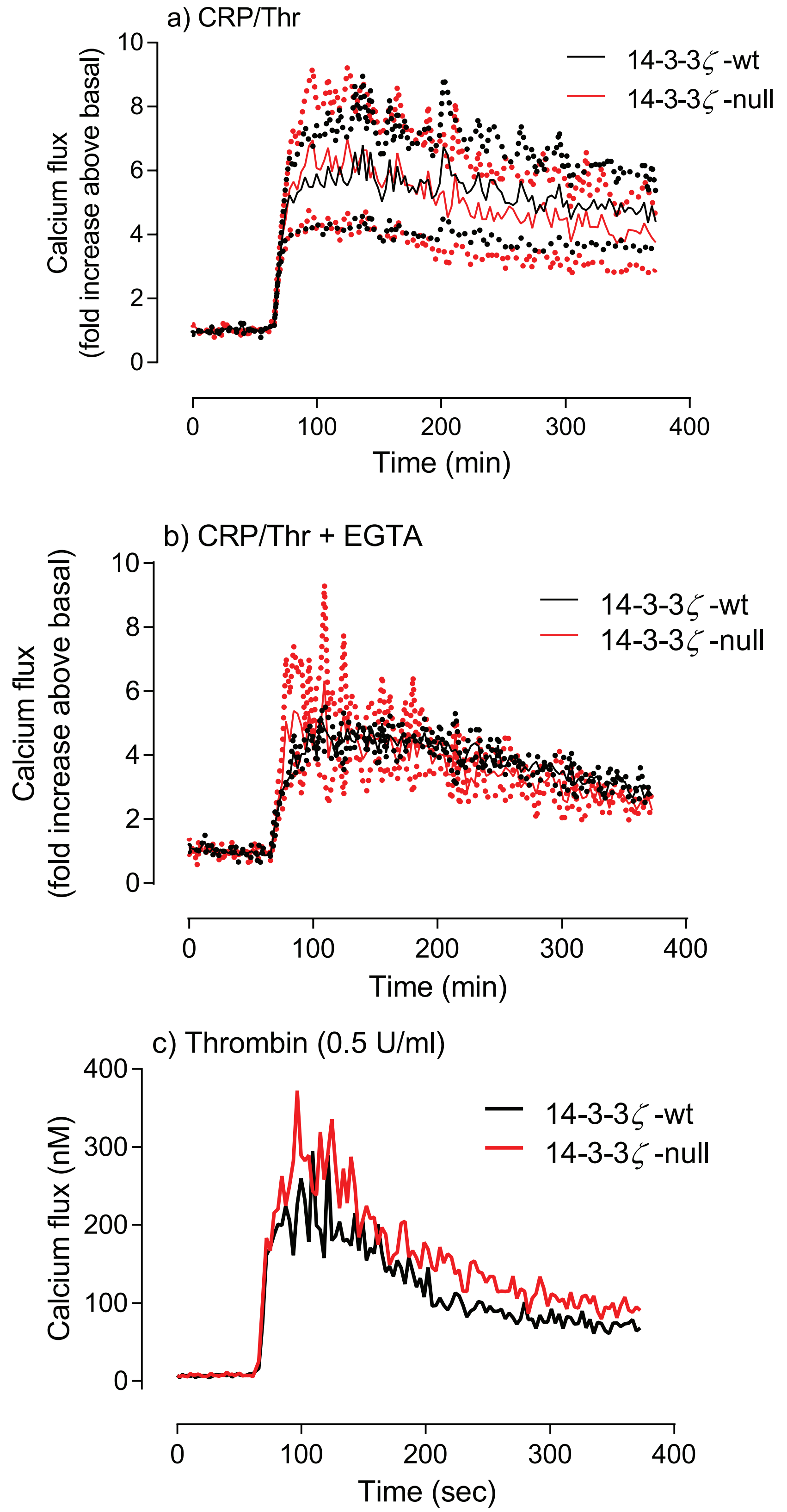

Supplementary figure 5.

Calcium flux in response to agonist is unchanged in 14 3 3ζ-deficient mice. Washed platelets isolated from 14-3-3ζ-wt (black) or 14-3-3ζ-deficient (14-3-3ζ-null, red) mice were loaded with calcium dyes and calcium flux measured over time using a ratiometric calcium assay, in response to CRP (10 μg ml^{-1})/thrombin (1.0 U ml^{-1}), in the absence (a) or presence (b) of EGTA (2 mM) or thrombin alone (0.5 U ml^{-1}) (c), as described under 'Methods'. Results depict the fold increase in calcium over basal (resting) level, and represent the mean ± SEM from 3 independent experiments, performed in duplicate. Statistical analysis using a 2-way ANOVA demonstrated no statistical significance over the time course between genotypes ($p>0.05$)

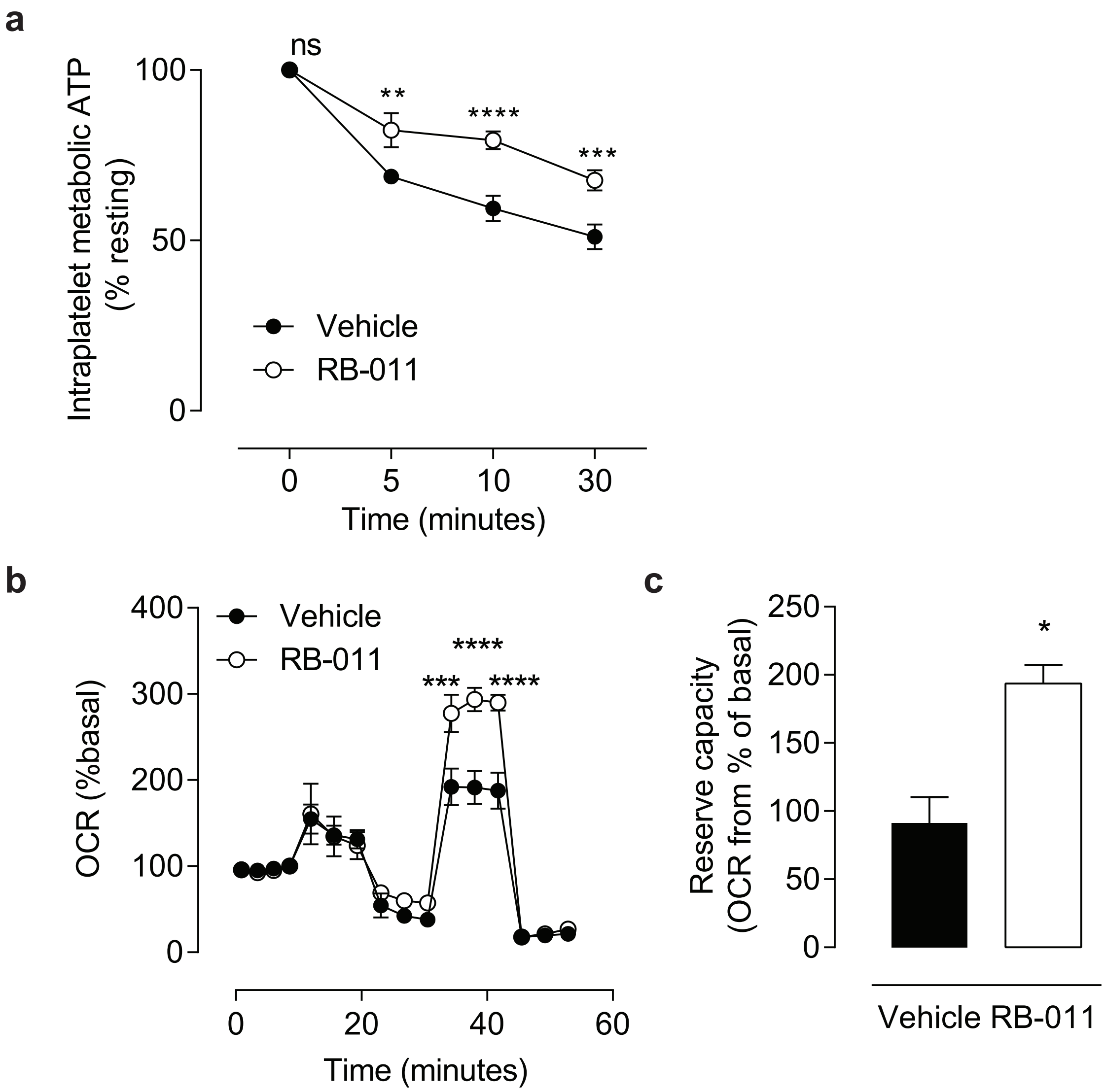

Supplementary figure 6.
RB-011 reduces metabolic ATP depletion and mitochondrial oxygen consumption rate. Washed platelets were isolated from anticoagulated whole blood from healthy volunteer donors and treated with a 14-3-3 dimer destabilizer (RB-011, 10 μM) or vehicle (sodium mesylate salt). Metabolic ATP (a) and oxygen consumption rate (OCR) (b) following stimulation with CRP/Thrombin were quantified, as described under "Methods". OCR has been depicted over the entire 60 minute time course (i), or as a specific change in reserve respiratory capacity, with % OCR increase over basal (ii). Results are expressed as the mean ± SEM (n=3), and analysed using a 2-way ANOVA (Bonferroni's post-hoc testing) where $^{ns}p>0.05$. These results indicate that treatment of human platelets with the 14-3-3 dimer destabilizer is consistent with the phenotype of 14-3-3ζ-deficient mouse platelets.

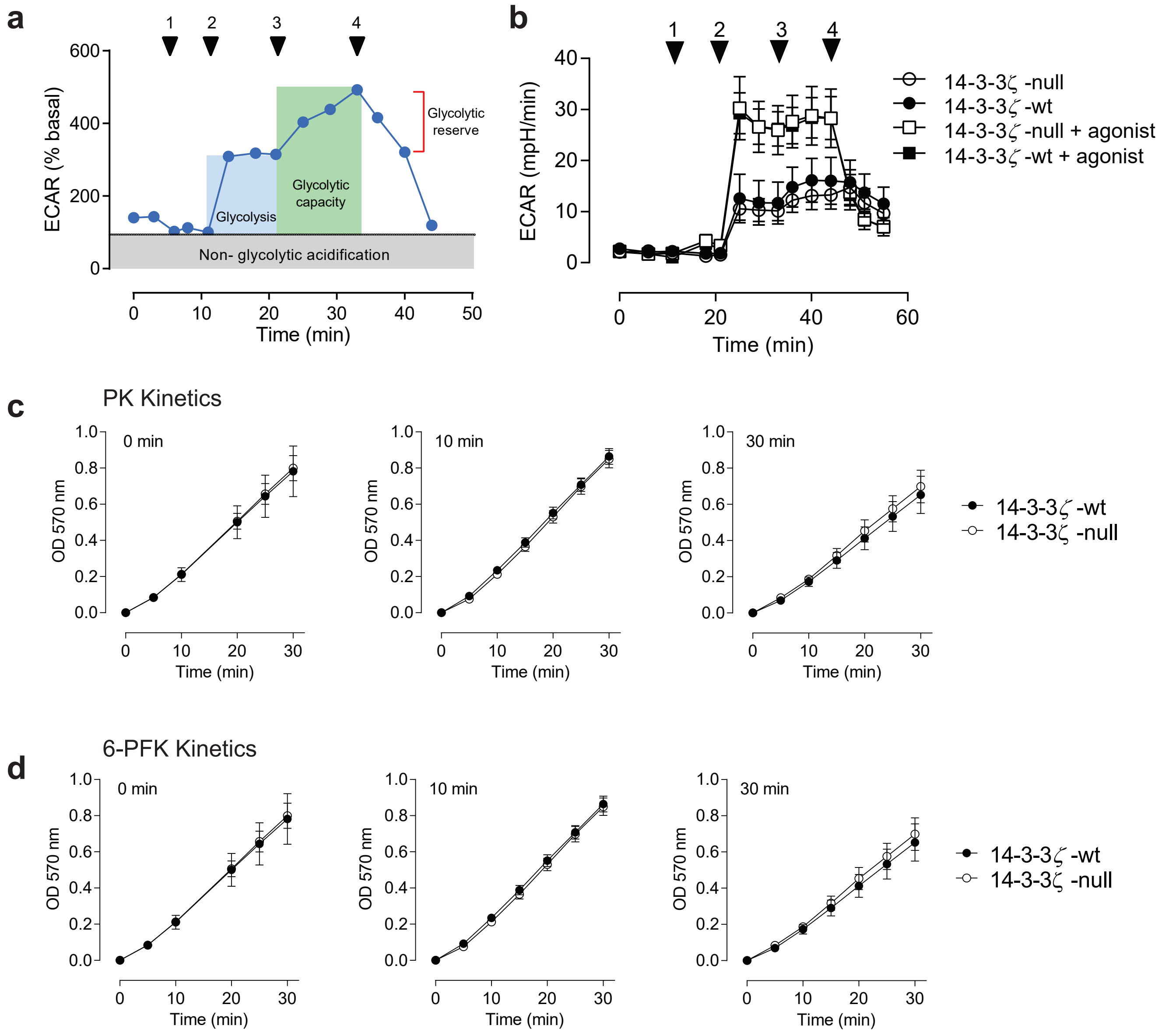

Supplementary figure 7.

Normal glycolytic capacity in 14 3 3ζ-deficient platelets. (a, b) Washed platelets were isolated from 14-3-3 ζ-wt (closed symbols) or 14-3-3ζ-deficient (14-3-3ζ-null) mice (open symbols). (a, b) Glycolytic capacity was measured in DMEM modified media using the Seahorse XFp analyser, according to manufacturer's instructions. (a) Representative trace identifying the typical pattern of ECAR, depicting basal level, glycolysis (blue), glycolytic capacity (green) and reserve capacity, following the injection of drugs including: 1) vehicle or agonist; 2) Glucose; 3) Oligomycin and 4) 2-DG. (b) Platelets were assayed for H^+ production in un-stimulated or CRP/thrombin-stimulated (0.25 µg ml^{-1}; 0.5 U ml^{-1}, +agonist)) conditions. (c, d) Pyruvate kinase (PK) (c) and 6-Phosphopyruvate kinase (6-PFK) (d) enzyme activity was measured in platelets treated with CRP/thrombin (0.25 µg ml^{-1}, 0.5 U ml^{-1}) for 0-30 min, as described in "Methods". Results depict kinetics of enzyme activity in 5 min increments, and represent the mean ± SEM (n=3). 2-way ANOVA statistical analysis (With Bonferroni's Post-hoc testing) was performed, with no statistical significance identified between 14-3-3 ζ-wt and 14-3-3ζ-deficient samples.

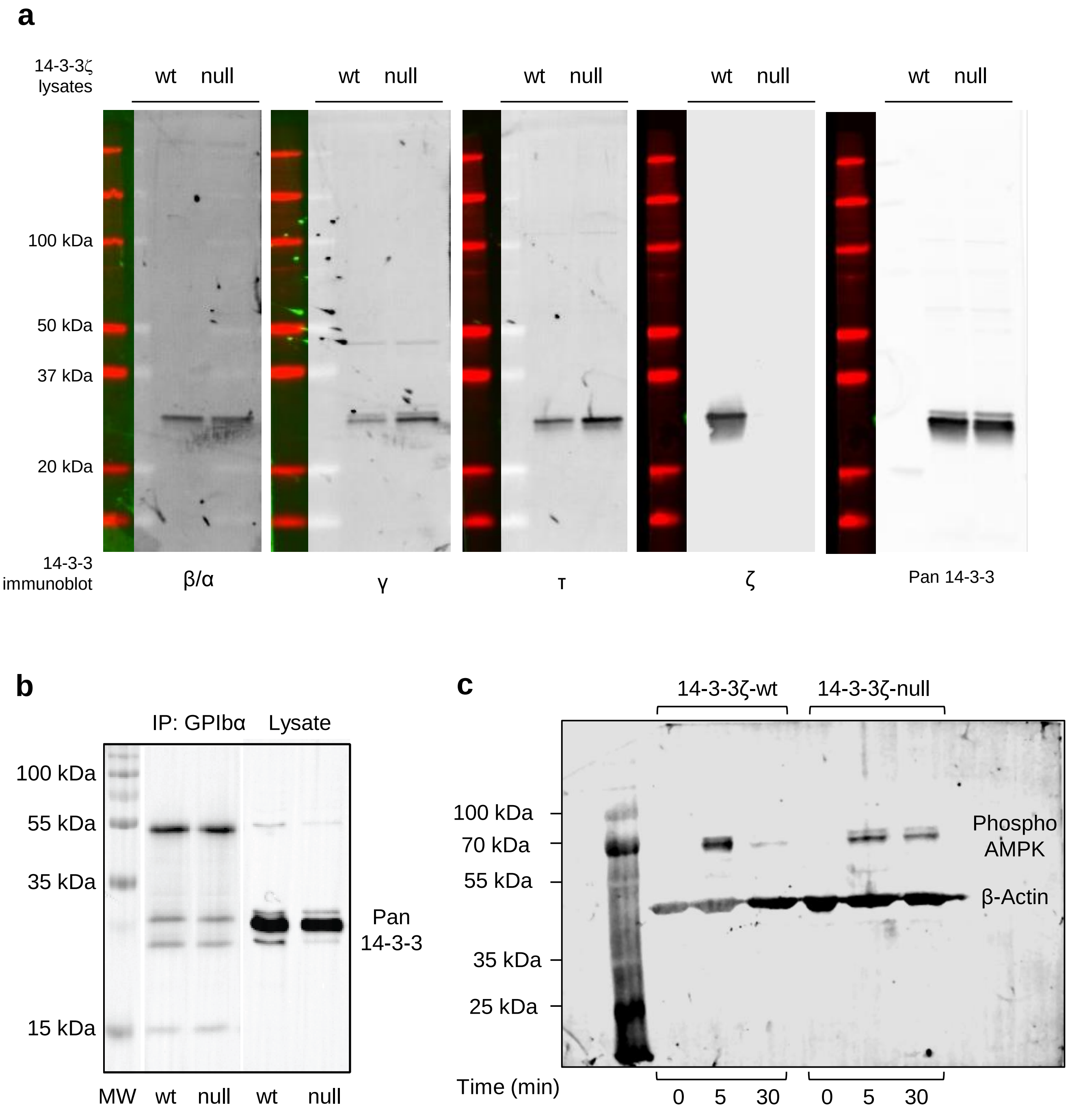

Supplementary Figure 8 - Original immunoblot data**.** Original uncropped immunoblot data used in Fig. 3g (**a**), Fig. 3i (**b**) and Fig. 6b (**c**).
